# A Custom Ultra-Low-Cost 3D Bioprinter Supports Cell Growth and Differentiation

**DOI:** 10.3389/fbioe.2020.580889

**Published:** 2020-11-04

**Authors:** Konstantinos Ioannidis, Rodolfos I. Danalatos, Spyridon Champeris Tsaniras, Konstantina Kaplani, Georgia Lokka, Anastasia Kanellou, Dionysios J. Papachristou, Georgios Bokias, Zoi Lygerou, Stavros Taraviras

**Affiliations:** ^1^Department of Physiology, School of Medicine, University of Patras, Patras, Greece; ^2^Laboratory of Bone and Soft Tissue Studies, Department of Anatomy-Histology-Embryology, School of Medicine, University of Patras, Patras, Greece; ^3^Department of Chemistry, University of Patras, Patras, Greece; ^4^Department of General Biology, School of Medicine, University of Patras, Patras, Greece

**Keywords:** 3D bioprinting, low cost 3D bioprinter, stem cell biofabrication, postnatal radial glial cells, bone-marrow mesenchymal stem cell, alginate-gelatin bioink

## Abstract

Advances in 3D bioprinting have allowed the use of stem cells along with biomaterials and growth factors toward novel tissue engineering approaches. However, the cost of these systems along with their consumables is currently extremely high, limiting their applicability. To address this, we converted a 3D printer into an open source 3D bioprinter and produced a customized bioink based on accessible alginate/gelatin precursors, leading to a cost-effective solution. The bioprinter’s resolution, including line width, spreading ratio and extrusion uniformity measurements, along with the rheological properties of the bioinks were analyzed, revealing high bioprinting accuracy within the printability window. Following the bioprinting process, cell survival and proliferation were validated on HeLa Kyoto and HEK293T cell lines. In addition, we isolated and 3D bioprinted postnatal neural stem cell progenitors derived from the mouse subventricular zone as well as mesenchymal stem cells derived from mouse bone marrow. Our results suggest that our low-cost 3D bioprinter can support cell proliferation and differentiation of two different types of primary stem cell populations, indicating that it can be used as a reliable tool for developing efficient research models for stem cell research and tissue engineering.

## Introduction

Three dimensional (3D) bioprinting is a new interdisciplinary research field, which utilizes computer engineering, material science, robotics and biomedical engineering in order to provide novel applications in life sciences through tissue engineering and regenerative medicine (Aljohani et al., 2018). By combining cells with biocompatible materials known as bioinks and by their precise deposition into desired structures, this new technology can be used as a tool for developing a variety of biological constructs for a versatile range of applications, including microfluidic devices such as organ-on-a-chip (Knowlton and Tasoglu, 2016), organoids from patient specific cells for high throughput drug development and precision medicine (Mazzocchi et al., 2019), more relevant study models for disease (Ma et al., 2018) or physiology (Madden et al., 2018) and tissue reconstitution suitable for partial organ transplantation (Noor et al., 2019). More importantly, 3D bioprinting offers the potential for customization, by allowing users to manipulate parameters such as biomaterial selection, cell type and 3D design, depending on the experimental setting. Furthermore, culture and differentiation of stem cells inside 3D *in vitro* systems generated by bioprinters (Hsieh et al., 2015; Bae et al., 2018; Sorkio et al., 2018) has attracted attention regarding the applications of 3D bioprinting in the field of regenerative medicine.

This technology can also provide significant advantages in biomedical research, mainly due to the fact that in certain cases *in vitro* research methods are been developed and leading to the replacement of animal models (Yun et al., 2018), thus reducing the cost and time needed for research. Additionally, 3D *in vitro* systems for drug screening show drug resistance due to the gradient soluble of chemical agents compared with 2D *in vitro* systems (Imamura et al., 2015), where all cells are exposed to the same concentration. For this reason, more reliable methods for drug development need to be established, which can reduce the cost and time of drug development by eliminating false selection of drug-hits obtained from 2D drug screenings.

It is therefore evident that widespread access to this new technology would benefit researchers in several different fields. However, current commercially available 3D bioprinters have a high cost (10,000–150,000$) and low customization capacity, while they also require costly consumables and highly skilled staff for operation and maintenance, limiting their applicability. In this regard, many researchers have tried to develop low cost 3D bioprinters based on different extrusion methods and materials (Mielczarek et al., 2015; Wang et al., 2015; Goldstein et al., 2016; Reid et al., 2016, 2019; Madihally and Roehm, 2017; Schmieden et al., 2018; Bessler et al., 2019; Kahl et al., 2019; Yenilmez et al., 2019). However, a 3D custom made bioprinter that is open source, ultra-low cost and easy to set up and operate, along with an evaluation of its applications for developing models in stem cell research, has not yet been reported.

In order to address this issue, we proceeded with the conversion of a desktop (FDM) 3D printer into a 3D bioprinter, according to the *DIYbio* movement approaches (Landrain et al., 2013). For the conversion, we first assembled the 3D printer and used it to 3D print the parts of a lightweight syringe pump unit and its mount on x-axis. We tried to keep the integration of the syringe pump into the 3D printer easy, by maintaining the stepper motor driver and the connection cables. Next, we decided to use a mixture of alginate and gelatin as bioink, mainly due to their biocompatibility and gelation properties (Chung et al., 2013; Axpe and Oyen, 2016; Li et al., 2018). The system integration and bioink selection were validated by performing resolution measurements along with rheological analyses and were followed by a case study of cell survival and proliferation on cell-laden bioprinted constructs of HEK293T and HeLa Kyoto cell lines. Additionally, in order to evaluate the impact of our chosen parameters on stem cell pluripotency and differentiation capacity, primary radial glial (pRGCs) stem cells, a neural stem cell population derived from mice subventricular zone and bone marrow derived mesenchymal stem cells were also 3D bioprinted and evaluated.

## Materials and Methods

### Bioprinter Assembly

An Anet A8 was assembled using a kit, according to manufacturer’s instructions. An open source syringe pump model from a previous study (Wijnen et al., 2014), was redesigned in order to attach to the moving x-axis of the printer. The STL file of the idler end of the syringe pump, that was redesigned in Tinkercad^1^ can be found at https://www.thingiverse.com/thing:3134313. We also designed a x-axis cartridge mount for the syringe pump unit, which can be found at https://www.thingiverse.com/thing:4491772. All 3D models of the components were converted into printable gcode instructions using the Slic3r software (GNU Affero General Public License). These parts were 3D printed using the aforementioned desktop 3D printer before its conversion, using the layer-by-layer method for the deposition of transparent Polylactic acid (PLA) filament (PrimaVALUE). The 3D printed parts used for the conversion of the 3D printer into a bioprinter can be found in Supplementary Figure S1. All printed parts, after being assembled (where needed), were integrated with the Anet A8, resulting in a functional 3D bioprinter.

### Animals

C57BL/6 wild type mice were used for the isolation of primary postnatal radial glial and mesenchymal stem cells. All procedures were performed according to the regulations of the Medical School of the University of Patras and were approved by the Achaia’s regional veterinary authority.

### Cell Lines and Primary Cultures

HeLa Kyoto and HEK293T cell lines were 2D cultured in Dulbecco’s Modified Eagle Medium (DMEM, Gibco) supplemented with 10% fetal bovine serum (FBS, Gibco; Thermo Fisher Scientific, Inc.). Postnatal day 0 (P0) radial glial stem cells (pRGCs) were isolated from the mouse subventricular zone and 2D cultured in proliferation medium (PM) containing DMEM F12-Glutamax (Gibco; Thermo Fisher Scientific, Inc.), 10% (v/v) FBS, 1% (v/v) penicillin and streptomycin (P/S, Gibco; Thermo Fisher Scientific, Inc.), Epidermal Growth Factor (EGF, Peprotech) and Fibroblast Growth Factor (FGF, Peprotech) at a final concentration of 5μg/ml each, as previously described (Lalioti et al., 2019). Primary bone marrow-derived mesenchymal stem cells (MSCs) were isolated from the mouse femur, as described previously (Amend et al., 2016). Cells were 2D cultured and purified in DMEM supplemented with 10% FBS and 1% P/S. All cells were cultured at 37°C in a humidified incubator with 5% CO_2_ and medium was changed every other day.

### Bioink Synthesis

All 3D bioprinting experiments were based on alginate-gelatin mixture as the bioink. For the HEK 293T and HeLa Kyoto cell lines, 1.8% (w/v) sodium alginate (C.E. Roeper) was dissolved in pre-warmed (37°C) DMEM (Gibco) and stirred overnight. Next, gelatin (Sigma) was dissolved in the alginate solution to a final concentration of 3% (w/v) and stirred at 40°C for 2 h. Specifically, a 0.8% (w/v) sodium alginate and 0.5% (w/v) gelatin bioink was synthesized for the pRGCs, while for the MSCs 2% (w/v) sodium alginate and 3% (w/v) gelatin was prepared as described above. In half of the MSCs samples Hydroxyapatite (HAP) was synthesized *in situ* after bioprinting.

### Bioprinting Process

Prior to the bioprinting process, a Computer-Aided Design (CAD) file for the printed construct was designed using Tinkercad (see footnote). The cells were mixed in a 3 mL leuer lock syringe with pre-warmed (37°C) bioink, at a final concentration of 2 ⋅ × 10^5^ cells/mL and incubated at 4°C for 30 min in order to achieve a printable viscosity. Next, the syringe was mounted into the syringe pump, while a 10 cm petri dish was fixed onto the moving bed with adhesive tape to be used as a printing platform. The bioprinting process was controlled by using the Printrun software^2^ through a computer connected to the USB port on the 3D printer’s mainboard. Post bioprinting, the cell embedded constructs were crosslinked with 2% (w/v) CaCl_2_, washed three times with Tris-HCl buffered saline (TBS) and then incubated into a six-well plate with the appropriate culture medium.

### Line Width, Spreading Ratio, and Extrusion Uniformity Ratio

The bioprinting procedure was evaluated for its accuracy and resolution. For this purpose, the extrusion rate was set to 20 mm/s with volumetric extrusion enabled through Slic3r settings, while the printing speed was set to 40 mm/s. Both were kept stable for all samples. The line width was measured by printing 5 layers, with a layer height set to 0.2 mm, and taking multiple measurements of the width of the printed lines for each bioink. The spreading ratio was then calculated by dividing the line width with the inner needle diameter (in this case a 25-gauge needle with 0.26 mm diameter). The extrusion uniformity ratio was measured as previously described (Gao et al., 2018). Briefly, printed lines were manually outlined on both sides, the length in pixels was measured and was then divided with the pixel length of a perfectly straight line. Finally, the influence of the printing parameters on the printed outcome was evaluated after producing different gcode instructions in the Slic3r program regarding travel speed, extrusion rate and the usage of two different nozzles (25G and 27G). For this analysis, the line width of the printed constructs was measured as described above. All measurements were performed using ImageJ v 1.50 (National Institutes of Health, MD, United States).

### Rheological Measurements

A Discovery Hybrid Rheometer 2.0 (TA Instruments, DE, United States) was used with a 20mm parallel plate geometry. The geometry gap was set at 200 μm for all the bioinks’ measurements. For all bioinks studied, an oscillatory strain sweep, ranging from 0.01% to 100% with an oscillating frequency of 1 Hz, was first conducted to determine the linear viscoelastic region (LVR; data not shown). Secondly, an oscillatory frequency sweep, ranging from 0.01 to 100 Hz and an oscillating strain fixed within the LVR, was performed to observe the change of storage modulus (G′) and loss modulus (G″). Bioinks for these tests were incubated at 4°C before taking measurements at 25°C, to mimic the bioprinting process. Next, the viscosity was evaluated by increasing the shear rate from 0.01 to 100 s^–1^. Finally, a temperature ramp for evaluating both G′/G″ and viscosity, was performed in separate measurements between 4 and 44°C for samples A1.8 G3 and A2 G3. The samples were first incubated at 4°C and were then allowed to equilibrate at the cooling plate (4°C) for 5 min before taking measurements. Loss tangent values (tanδ = G ″/G′) were generated from an oscillatory frequency sweep, as described above.

### Cell Viability

The percentage of cell viability was determined using Trypan blue on HeLa Kyoto cells. The percentage of positive (dead) cells was measured with a hematocytometer. Cells were incubated for 30 min at different temperatures (4 and 37°C) after been mixed within the bioink, consisting of A1.8 G3. For releasing the embedded cells, 0.1 M sodium citrate (pH = 7.4) was used as a chelating agent at 37°C until the gel was dissolved, as previously described (Nemati et al., 2019). In the control group (C), cell viability was measured without the 30 min incubation step and mixture with bioink.

### 3D Cell Culture

HEK 293T, HeLa Kyoto and pRGCs bioprinted constructs were cultured using the same culture medium, as in 2D cultures prior to mixing with bioink. For MSCs, hydroxyapatite (HAP) crystals were produced inside the alginate-gelatin matrix by *in situ* calcification of the bioprinted scaffold using sequenced incubation with culture medium enriched with 0.05 M CaCl_2_ for 10 min and accordingly with 0.03 M Na_2_HPO_4_. The proliferation medium used for MSCs after bioprinting was DMEM F12 supplemented with 15% (w/v) FBS, 1% (v/v) P/S, 0.126% (w/v) NaHCO_3_ and 1.25% (v/v) L-glutamine. In all experiments the medium was changed every other day.

### Alkaline Phosphatase Assay

To assess the alkaline phosphatase (ALP) activity, staining was performed in both 2D and 3D cultures. Samples were fixed with cold 10% Neutral Formalin Buffer for 15 min. After rinsing with dH_2_O, samples were incubated for 45 min in room temperature with 0.1 mg/ml Naphthol AS-MX phosphate (Sigma) substrate in 0.4% N, N-dimethylformamide and 0.1M HCl, pH 8.3 with 0.6 mg/ml fast red violet LB salt (Sigma) to visualize the product. The staining solution was washed away with dH_2_O. The products of enzyme activity witnessed as red stains indicate the ALP activity and were observed under the stereoscope (Leica MZ 16F). ALP staining was quantified using ImageJ v1.50 by measuring the percentage of red stained area vs. total area in each image.

### qPCR Analysis

Samples from 2D and 3D cultures were frozen at −80°C in Trizol reagent (Ambio) and RNA was extracted according to manufacturer’s instructions. cDNA was synthesized using the M-MLV reverse transcriptase (Invitrogen) and qPCR was performed with the Kapa SYBR Fat qPCR kit (KapaBiosystems) using quantitative real−time PCR (Applied Biosystems StepOne), as described previously (Arbi et al., 2016). The primers used for Runx2, Osteopontin, Collagen Type 1 and Alkaline Phosphatase are shown in Supplementary Table S1. Samples were normalized using GAPDH. All data shown were analyzed in technical duplicates and one biological replicate. REST-MCS beta software was used for qPCR data analysis.

### Immunofluorescent Staining

Immunofluorescence was performed as described previously (Stathopoulou et al., 2012; Dimaki et al., 2013; Iliou et al., 2013; Champeris Tsaniras et al., 2018). Samples at day 1 and at day 15 were fixed in 4% paraformaldehyde for 20 min at 37°C. 0.5% Triton X detergent was then used to permeabilize the cells. Prior to staining with primary antibodies, samples were blocked with 3% (w/v) BSA in FBS. Samples from HEK and HeLa timepoints were stained with 1:1,000 anti-rabbit Ki67 (Zytomed, 2705) and 1:1,000 anti-mouse α-tubulin (Sigma, T8203) for 24 h, while samples from pRGCs were stained accordingly with 1:1,000 anti-mouse IgG1 Pax6 (DSHB), 1:1,000 anti-rabbit Sox2 (Abcam, ab97959) and 1:1,000 anti-rabbit GFAP (Dako, z0334), Ki67 (Zytomed, 2705) at day1 timepoint and at day15 timepoint were stained with 1:1,000 anti-rabbit GFAP (Dako, z0334) and anti-mouse α-tubulin. Samples were then stained with secondary fluorophore antibodies and Hoechst (Sigma). Cell morphology and proliferation was evaluated for the whole mounted samples inside imaging dishes (Ibidi, μ-Dish 35 mm) using a confocal microscope (Leica TCS SP5).

### Statistical Analysis

In all samples, normality was verified using the D’Agostino-Pearson test. Line width, spreading ratio, extrusion uniformity ratio and cell viability were compared using one-way ANOVA followed by Bonferroni correction. ALP quantification values were compared using *t*-test. Analyses were performed by GraphPad Prism v8 (CA, United States).

## Results and Discussion

In this report, we demonstrate the conversion of a low cost commercially available 3D printer (∼120$) into a 3D bioprinter. In addition, the bioinks used in this study are based on alginate-gelatin, thus resulting in low cost polymer precursors that are available at any biomedical laboratory. After the 3D bioprinter’s conversion and bioink’s selection, we analyzed both the bioprinter’s accuracy and bioinks’ rheological properties. Subsequently, we demonstrated high cell survival and cell proliferation for HEK293T and HeLa Kyoto bioprinted 3D cultured cell lines, even in late culture timepoints (day 15). Although, these cell lines are commonly used as a reliable model, certain applications require more specific cell types, such as primary stem cells. Therefore, to investigate whether our custom made bioinks can maintain cell differentiation and self-renewal capacity of the embedded primary derived stem cells, pRGCs were isolated, 3D bioprinted and cultured for 2 weeks until immunofluorescent analysis. After validating the 3D bioprinted cultures of pRGCs, regarding their behavior in 3D, we wanted to investigate another stem cell population (MSCs), which exhibit mechanosensitivity and hence their differentiation can be altered depending on the stiffness of their microenvironment (Freeman and Kelly, 2017; Liu et al., 2020). To this end, we validated that primary bone marrow derived mesenchymal stem cells were able to differentiate into osteoblasts, after being 3D bioprinted, with or without the addition of HAP.

### Bioprinter Assembly and Bioprinting Process

Novel research methodologies, for a broad range of applications, can be established by using a 3D bioprinter in biomedical research. Thus, by converting a desktop 3D printer to a 3D bioprinter and by producing a customade bioink, we showed that, this technology can be democratized for every scientist by overcoming the high cost of commercially available systems and consumables. For this reason, we selected to start with a basic desktop 3D printer that is low cost, reliable and highly accurate at the same time. The obtained 3D printer (Anet A8 Prusa i3 DIY kit) is easy to set up and calibrate within a working day, according to the manufacturer’s instructions.

**FIGURE 1:**
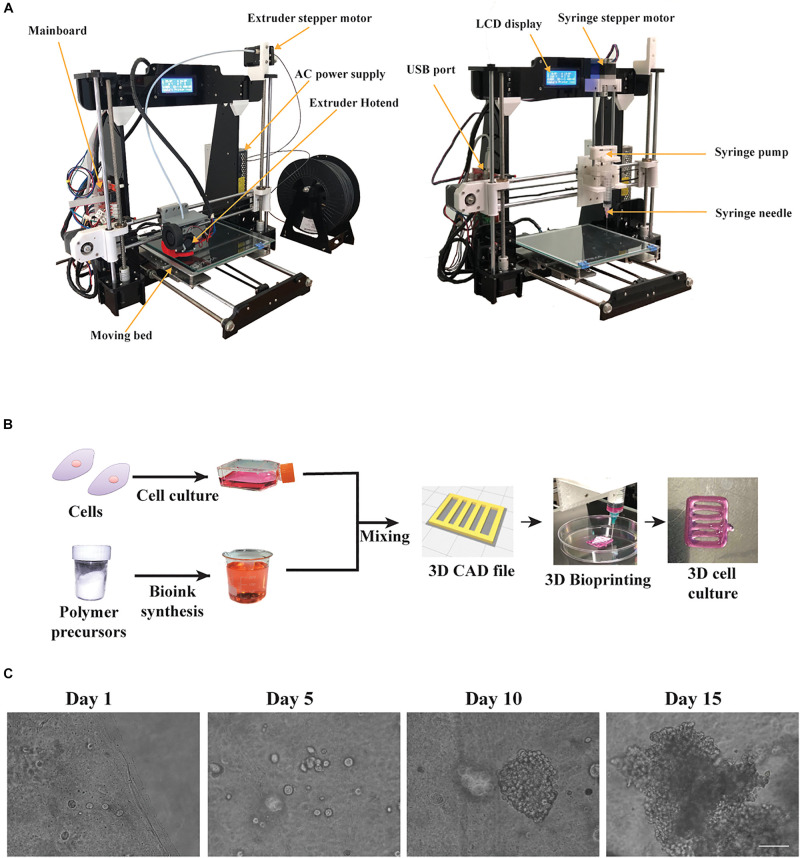
The bioprinting process using our custom made bioprinter. **(A)** The 3D desktop 3D Anet A8 printer before the conversion equipped with an extruder hotend and the modified 3D bioprinter equipped with an open source, 3D printed syringe pump along its 3D printed mount on x-axis. **(B)** The bioprinting process: cells were cultured in 2D until mixed with the custom bioink, to fabricate a 3D construct, following the digital blueprints, embedded with HeLa cells. **(C)** Phase contrast microscope images showing cluster formation of the embedded HeLa cells for different timepoints. Scale bar set to 320 μm.

In order to facilitate the controllable extrusion of cell laden bioinks, an open source syringe pump unit from a previous study was partially redesigned (Wijnen et al., 2014), facilitating the connection on a 3D printed x-axis mount of the syringe unit, that was originally designed by us (Supplementary Figure S1). Both the syringe unit and it’s x-axis mount were 3D printed using PLA, resulting in a lightweight construct. In addition, in order to keep the conversion easy without many hardware changes, we maintained the major 3D printer’s components, such as the stepper motor and the connection cables. This ensures firstly, that the system would not require additional changes in the firmware used to control the printing process and secondly, that the system can be converted by anyone without any special knowledge. The 3D printed parts of the syringe unit were then assembled as described previously. Thereafter, the syringe unit was connected to the x-axis mount and then integrated with the 3D printer’s mainboard (Figure 1A). Moreover, configuration of the set up can be adjusted according to the application, because the system is open source and thus additional upgrades could be 3D printed and integrated into the configuration. Therefore, because every upgrade is 3D printed, our 3D bioprinter after the conversion resulted in a low weight portable device suitable for use in a laminar airflow chamber. In addition, the syringe unit can carry any type and volume of available syringes and needles, but in our case, we used single use 5 or 10 mL luer lock syringes along with single use luer lock needles (25G). This ensures that the bioprinting process, would take place with sterile conditions ensuring no contamination into the cell culture.

**FIGURE 2:**
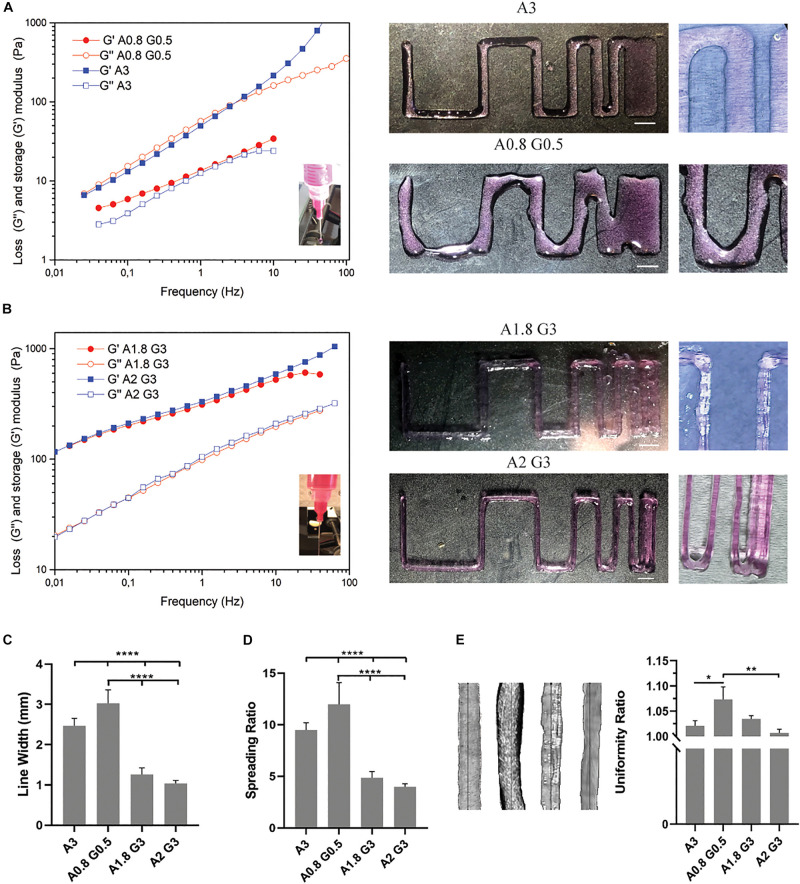
Bioprinting resolution analysis of bioinks and evaluation of bioprinting parameters. **(A)** Rheological analysis and representative macroscopic images of the bioprinted viscoelastic liquid bioink consisting of alginate 0.8%/gelatin 0.5% (A0.8 G0.5) and alginate 3% (A3). **(B)** Rheological analysis and representative macroscopic images of the bioprinted viscoelastic gel bioinks consisting of alginate 1.8%/gelatin 3% (A1.8 G3) and alginate 2%/gelatin 3% (A2 G3). **(C)** Histogram showing the line average width (in mm) of the bioprinted lines from **(A,B)**, for the evaluation of bioink printability, showing the representative resolution and reproducibility of all bioinks used in the study. **(D)** Spreading ratio determined from line width divided by the inner nozzle diameter. **(E)** Macroscopic images of bioprinted lines from all bioinks used for quantification of the extrusion uniformity. Scale bars represent 5 mm. All experiments were conducted in triplicate (*n* = 3). Values are mean ± *SD*. *p < 0.05, ***p < 0.001, and ****p < 0.0001.

The bioprinting process, as shown in Figure 1B, consists of the prior bioprinting phase, where alginate/gelatin bioinks were synthesized and suitable cells were selected. The alginate/gelatin bioinks were produced in different concentrations, based on the cell line used, the printability and the cell viability reported on previous studies (Mondal et al., 2019; Chawla et al., 2020). Next, after cells were cultured in 2D conditions until confluency, they were mixed with the appropriate bioink and were bioprinted according to the gcode instructions, generating the 3D culture design shown in Figure 1B. Then, the bioprinted cell laden constructs, after being crosslinked with CaCl_2_, were cultured for 2 weeks. The cells appeared as single spheroids in early culture timepoints and through the progression of time in culture they started forming increasingly larger clusters, as observed under light microscopy (Figure 1C). Previous studies (Li et al., 2011; Cidonio et al., 2019) suggested that the syringe’s diameter and the needle’s diameter play a critical role regarding the printing outcome. For this reason, diameter values (mm) of inner syringe’s diameter and inner needle’s diameter were incorporated into the Slic3r program, which is used for producing the gcode instructions. In our case, a 5 mL luer lock syringe along with a luer lock needle (25G) produced the best replicas of the CAD file in terms of dimension consistency and reproducibility, and were also verified through measurements of the average line width, spreading ratio and extrusion uniformity (Figure 2). In addition, we investigated the influence of different printing parameters such as travel speed, extrusion rate, and nozzle diameter (Supplementary Figure S6). These measurements showcase the ideal printing parameters (travel speed ranging from 20 to 60 mm/s, extrusion rate ranging from 20 to 60 mm/s and use of a 25G nozzle) tested on the most viscus bioink (A2 G3) in our study. In the case of the 27G nozzle, our system could not provide the required higher pressure for the extrusion, hence the printing outcome was under extruded in all tested parameters. Lastly, the viscoelastic nature of the bioinks causes a small amount to be extruded even if the extrusion command is over. To overcome this, a retract motion for the syringe’s pump stepper motor had to be added to the slicer program to provide a gcode file, which could produce a negative pressure at the syringe tip. In this regard, the value for retraction distance was set to 7mm with a retraction speed of 25 mm/s. A more accurate, albeit more expensive bioprinting method for eliminating such problems would be pneumatic based extrusion. A video showing the bioprinting process is available at Supplementary Video 1.

### Bioprinter Resolution and Rheological Properties of Bioinks

Subsequently, we conducted rheological measurements which showed that the bioink consisting of 0.8% alginate and 0.5% gelatin, sample A0.8 G0.5, had similar viscoelastic characteristics (G″ and G′ values are close) with the 3% alginate sample (sample A3, used as a control to examine the difference of the bioink’s behavior in the absence of gelatin). Alginate has been reported (Gao et al., 2018) as a viscoelastic liquid in all concentrations, while gelatin has the most viscoelastic solid-gel behavior (G′ dominates; G′ > G″). As a consequence of the synergistic effect of the combination of the two materials at these low concentrations, sample A0.8G0.5 is similar to sample A3, however somewhat more viscous. In Figure 2B, the rheological measurements of the bioinks, consisting of 1.8 or 2% alginate and 3% gelatin, samples A1.8 G3 and A2 G3, revealed the impact of gelatin’s thermosensitive partial crosslinking, which contributed to a synergetic effect observed by the increase of G″, derived from the alginate fraction, and mostly the pronounced enhancement of the G′ value, derived from the gelatin fraction. These bioinks preserved their viscoelastic gel-like behavior when mimicking the environmental conditions of the bioprinting process during the rheological studies. They are thus suitable for an accurate bioprinting procedure where the average line width, spreading ratio and extrusion uniformity were evaluated (Figures 2C,E). In these measurements, the A0.8 G0.5 and A3 bioinks showed highly significant differences compared to all other formulations, mainly due to their less viscous nature. On the other hand, they could produce less shear stress to the embedded cells as less extrusion pressure was required; this makes them desirable candidates for bioprinting delicate cells, such as pRGCs. Ultimately, such bioinks could have better shape fidelity results, if they were bioprinted in a support bath as previously described (Jiang et al., 2017). In our cell culture experiments, they were only bioprinted at a height of 3 layers, to minimize the spreading ratio and preserve the extrusion uniformity.

### Cell Survival and Proliferation After the Bioprinting Process

**FIGURE 3:**
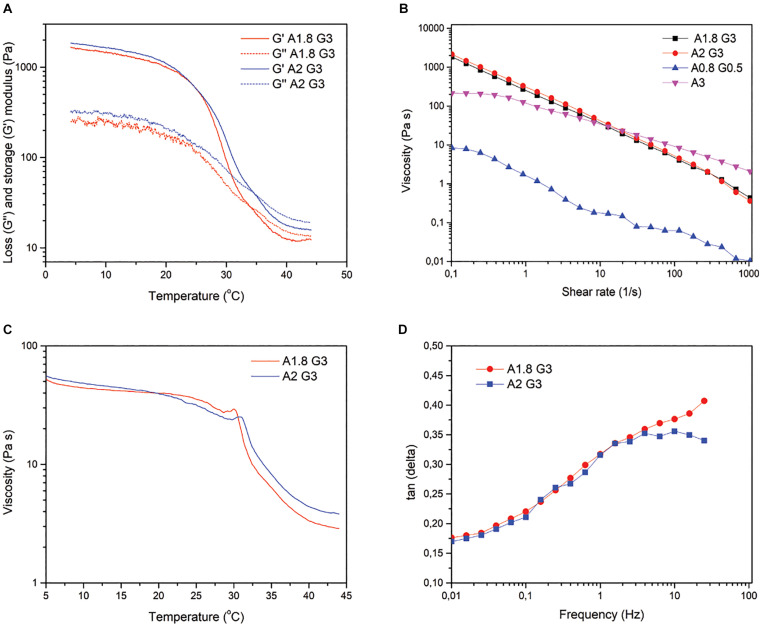
Rheological properties of Alginate 1.8/Gelatin 3% and Alginate 2%/Gelatin 3%. **(A)** Temperature-controlled rheological behavior of A1.8 G3 and A2 G3, showing the storage modulus (G′) and loss modulus (G″) as a function of temperature at a constant 10% strain and frequency of 1 Hz. **(B)** Viscosity of all the bioinks at different shear rates for comparison with A1.8/2 G3. **(C)** Viscosity profile of the bioinks as a function of temperature at a constant shear rate of 10 s^–1^. **(D)** Tangent δ as a function of frequency measured at 25°C with an oscillatory 10% strain.

**FIGURE 4:**
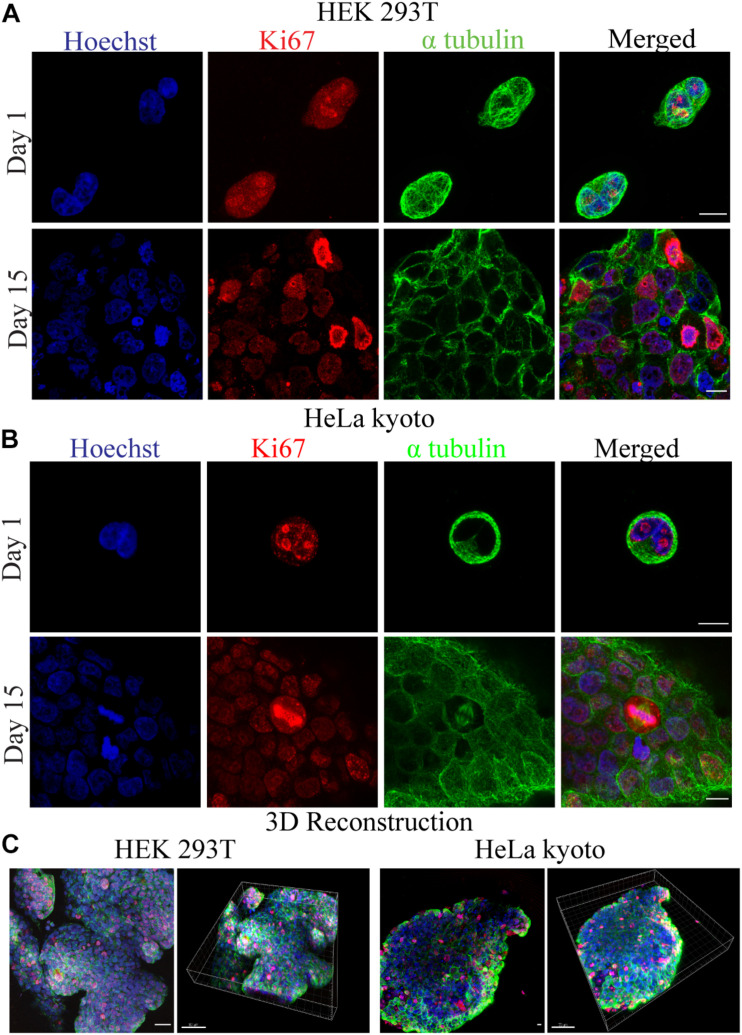
Confocal fluorescence microscopy images for the investigation of cellular viability and morphology. **(A)** HEK 293T and **(B)** HeLa Kyoto cells were embedded in 1.8% alginate/3% gelatin bioink and stained for Ki67(red), α-tubulin (green), and Hoechst (blue) at day 1 and 15. **(C)** Maximal projection confocal images of several focal planes along with their 3D reconstructions, for HEK 293T and HeLa Kyoto cells, accordingly.

Following the bioprinter’s calibration, we synthesized bioinks based on low cost polymer precursors (alginate, gelatin). As shown in Figures 2, 3, these natural polymers, when combined, apart from their known biocompatibility, possess special crosslinking and rheological properties (Mondal et al., 2019; Figures 2, 3). Prior to bioprinting, cells were first mixed into the syringe with warmed (37°C) bioink solution, which lowers the viscosity and thus allows cells to mix efficiently, as shown by the oscillatory temperature ramp of A1.8 G3 and A2 G3 in Figures 3A,C. In addition, these bioinks were found within the printability window (tanδ = 0.25–0.45, (Gao et al., 2018) as observed in Figure 3D, with tanδ = 0.32 at 1 Hz. Next, the cell laden bioink solution was incubated at 4°C for 30 min, hence the solution could undergo proper gelation to ensure printability (Figure 3A). In order to show that no cell loss was present due to incubation at 4°C for 30 min, we performed cell viability quantification on HeLa cells embedded in A2 G3, which was the bioink with the highest G′ in our study. In contrast to a previous study (Zhao et al., 2015), our bioink composition and incubation time did not affect significantly the cell viability prior bioprinting (Supplementary Figure S5). Secondly, to prove that cells were proliferating, clusters from HEK 293T and HeLa cells were immunostained against α tubulin and Ki67 for confocal microscopy analysis on different culture timepoints (Figures 4A,B). Our analysis shows that cells expressing different levels of Ki67, were observed at day 1 and 15 suggesting that our conditions maintain their proliferating capacity. A 3D reconstruction of the images obtained on day 15 shows that the center of the cluster does not contain Ki67 expressing cells, indicating a possible inhibition of the cell cycle due to contact inhibition inside the cluster (Figure 4C and Supplementary Figure S2). Regarding their morphology as assessed by α-tubulin, cells appeared as spheres due to lack of adherent proteins in the bioink (timepoint day 3 Supplementary Videos 2A,B and timepoint day 10 Supplementary Videos 3A,B), which is in agreement with previous studies (Ouyang et al., 2015; Jiang et al., 2017).

### Self-Renewal and Differentiation Potential of Primary Derived pRGCs

**FIGURE 5:**
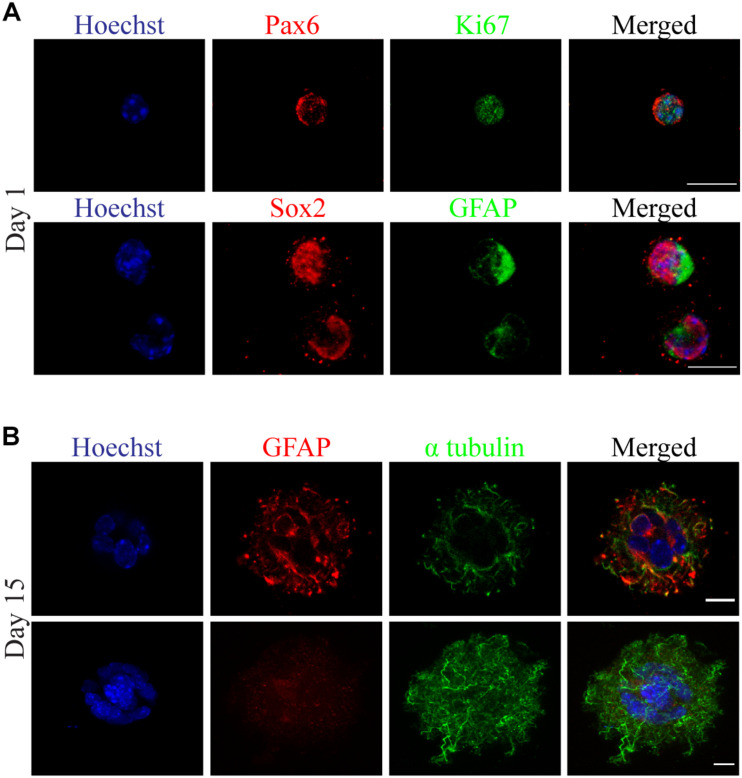
Neurosphere formation observed with confocal florescence microscopy images of 3D cultured primary radial glial cells (pRGCs) derived from postnatal mouse subventricular zone. **(A)** Cultured pRGCs were embedded in 0.8% alginate/0.5% gelatin bioink and after bioprinting they were stained with antibodies against Pax6 (red), Ki67 (green) or Sox2 (red), Gfap (green) and Hoechst (blue) at day 1. **(B)** Heterogeneity of 3D cultured pRGCs indicated by Gfap (red), α-tubulin (green), and Hoechst (blue) staining at day 15. Scale bars represent 10 μm.

In addition, we established a new series of experiments for the validation of self-renewal and differentiation capacity of primary mouse derived neural stem cells, bioprinted in 3D cultures. We isolated postnatal radial glial progenitors (pRGCs) derived from the subventricular zone (SVZ) (Kyrousi et al., 2015) of newborn (P0) mice, which can give rise to different subpopulations, in order to address their behavior post bioprinting in a proliferating culture. These cells are very delicate, as previously evaluated from *in vitro* 2D cultures (Kyrousi et al., 2015). To this regard, we preferred to use a low viscosity bioink (Figure 3B), which will produce less shear stress, while in addition, it will reflect the brain matrix stiffness as several other studies have proposed (Crompton et al., 2007; Moxon et al., 2019; Distler et al., 2020). Following the bioprinting process, cultures were maintained in self-renewal conditions and subsequently were stained using antibodies recognizing Pax6, Sox2, GFAP, and Ki67. Different subpopulations of cells were identified by the expression of the neurogenic transcription factor Pax6 and self-renewal and pluripotency marker Sox2 (Figure 5A). An increased number of proliferating progenitors with neurogenic fate was identified in cells maintained in 3D cultures at day1. Similarly, neurospheres which were formed in later time points (day 15) were immunostained using antibodies against GFAP and α-tubulin. These findings indicate the presence of different subpopulations, including astrocytes (GFAP +) and cells of neural lineage (Pax6 +) (Figure 5B). The same cellular subpopulations were also verified by immunostaining using specific antibodies recognizing Sox2, GFAP and α-tubulin in the same timepoint (Supplementary Figure S4). Compared with current established protocols for 3D neurosphere formation in low-adherent plates (Zhou et al., 2016), the method we used was cost- and time-effective, easy and automated for high throughput experiments. Regarding the choice of bioink, we used 0.8% alginate with 0.5% gelatin; this required less extrusion pressure; hence the shear stress was kept to a minimum. Neurosphere 3D reconstructions are shown in Supplementary Figure S3 and Supplementary Video 4.

### Scaffold Induced Osteogenic Differentiation of Bone-Marrow Derived Mesenchymal Stem Cells

**FIGURE 6:**
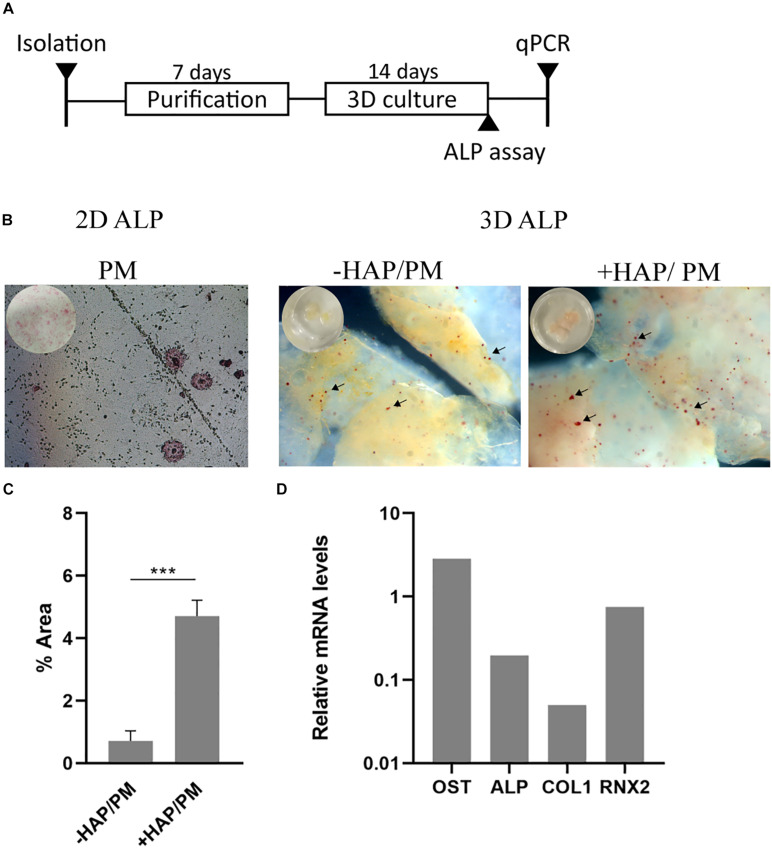
Osteogenic 3D differentiation of mesenchymal stem cells derived from mouse bone marrow. **(A)** Schematic illustration of the experimental timeline. **(B)** Alkaline phosphatase assay conducted on 2D and 3D cell cultures. In 2D culture, cells were cultured with proliferation medium (PM). In 3D culture, cells were cultured with PM together with the addition of hydroxyapatite (+ HAP/PM), which was formed *in situ* within the 3D bioprinted scaffold, or PM alone (–HAP/PM). **(C)** Quantification of the percentage of the alkaline phosphatase area in 3D cultures (+ HAP and –HAP; *n* = 3). **(D)** RT-qPCR of four osteogenic related genes (Osteopontin, Alkaline Phosphatase, Collagen Type I, and Runx2) from + HAP/PM after 3 weeks of culture in PM. Gene expression is relative to the expression of mRNA levels from sample 2D/PM (*n* = 1). ***p < 0.001.

In order to study the impact of the bioink on mesenchymal stem cell differentiation into osteoblasts, we conducted a series of experiments in which *in situ* HAP formation, was induced post-bioprinting by treating the bioprinted cell laden constructs with solutions of CaCl_2_ and Na_2_HPO_4_, as described previously (Szatkowski et al., 2015). It is known that mesenchymal stem cells are mechanosensitive (Xiao et al., 2016), therefore changes in the mechanical properties of their microenvironment may lead to changes of their behavior as well. We have therefore decided to use bioink consisting of 2% alginate and 3% gelatin, which as shown from Figures 2B, 3B that has high storage modulus and viscosity; hence facilitating the desired osteogenesis of the embedded cells over adipogenesis previously suggested from other studies (Freeman and Kelly, 2017; Liu et al., 2020). In addition it has been previously shown that HAP enhances the osteogenic differentiation of mesenchymal stem cells as shown also from other studies (Viti et al., 2016). Subsequently, we investigate whether the MSCs were differentiating due to the impact of the 3D scaffold and whether this effect was further enhanced by HAP presence. To this end, the 3D bioprinted cultures of primary mesenchymal stem cells derived from mouse bone marrow were cultured for 3 weeks in proliferation medium. The reference condition used in our experiment, was MSCs cultured in 2D petri dish with proliferation medium for the same culture period as the 3D bioprinted samples (Figure 6A). After the culture period both in 2D and 3D cultures, the skeletal alkaline phosphatase (ALP) was assessed enzymatically in order to determine the presence of generated osteoblasts. Our results show the presence of several alkaline phosphatase positive cells (Figure 6B). By quantifying the total alkaline phosphatase area in 3D samples (-HAP/ + HAP), we observed a significant increase in ALP intensity upon addition of HAP (Figure 6C), suggesting that our 3D culturing conditions support the osteogenic differentiation of MSCs. In addition, we provide a molecular analysis based on the relative expression of mRNA levels of four osteogenic related genes (Osteopontin, Runx2, ALP, and Collagen Type I) between 2D/PM and HAP/PM, supporting the osteogenic nature of the samples (Figure 6D). In addition, these data suggest that the + HAP/PM compared to the 2D sample has more mature osteoblasts, indicated by the downregulation of ALP and Collagen Type I genes, which are upregulated when osteoblasts are in a premature stage (Huang et al., 2007).

### Comparison to Previously Published DIY Bioprinters

Regarding the applicability of 3D bioprinting, several DIY bioprinters have already been described. One of the first studies was performed by Mielczarek et al. (2015), who presented only a prototype without performing any cell culture experiments. Soon thereafter, Goldstein et al. (2016) turned a desktop 3D printer (the MakerBot Replicator^®^ from MakerBot Industries, NY, United States) into a bioprinter; however, the cost of this system was not cheap as the MakerBot alone costs ∼2,000$ while the printing resolution and cell culture experiments were not sufficiently characterized in this study. Another study obtained better printing resolution using a hybrid bioprinter with both inkjet and extrusion print heads; however, this printer costs $1,370 and is too complicated to be replicated as it was based on a three axis CNC machine, a custom-made controller and DIY mechanical parts (Yenilmez et al., 2019). Newer studies have used the Prusa i3 (Bessler et al., 2019) and the Felix 3.0 3D (Reid et al., 2016, 2019) printers to increase the printing resolution; these printers cost ∼850$ and ∼$1,700, respectively, before their conversion. The latter achieved impressive single-cell resolution using a micro stepper motor and micropipette; however, the system comes at an overall final cost of ∼1,900$ (Reid et al., 2016).

Our system, together with the bioprinter by Kahl et al. (2019) published last year are the only ultra-low-cost bioprinters available. One of the novelties in our system is the modified syringe pump which can fit syringes of different sizes and allows for syringes to be plugged in and out in an instant. This is of essence due to the time-sensitive nature of the bioprinting process when working with cells. In contrast, the syringe pump by Kahl et al. (2019) has fixed positions for the syringe holder and changing the syringe is time-consuming. In addition, the cost of our bioprinter is extremely low (∼$230), making it one of the cheapest available while retaining significant printing resolution. In addition, our bioprinter has been extensively tested for its ability to bioprint several cell lines and delicate neural stem cells (pRGCs), reported here for the first time. Finally, mesenchymal stem cells (MSCs) were able to survive the bioprinting process, to proliferate and we enhanced their differentiation into osteoblasts by the introduction of *in situ* formatted HAP within the bioinks’ matrix.

## Conclusion

In this report, we provide detailed instructions for the modification of a commercial 3D printer into an ultra-low-cost 3D bioprinter. Both the rheological properties of the bioinks used and the bioprinter’s accuracy were examined, indicating a high accuracy for the 1.8/2% alginate and 3% gelatin mixtures. We showed that the bioprinter’s accuracy is heavily dependent on the bioinks’ rheological behavior; thus, the 0.8% alginate with 0.5% gelatin showed a moderate bioprinting accuracy at high layer heights, due to its lower viscosity and viscoelastic liquid nature. In addition, 1.8/2% alginate with 3% gelatin possessed acceptable rheological properties showing high bioprinting accuracy and resolution and used for 3D cell culture efficiently supporting cell growth both for established cell lines, neural and mesenchymal stem cells. These models are supporting self-renewal and differentiation of stem cell populations mimicking the *in vivo* processes. Thus, our 3D bioprinter can be used for developing new models to study human physiology and pathophysiology with an affordable cost. Further development on bioink composition and enrichment with extracellular matrix molecules (Ahn et al., 2017) can improve the ability to recapitulate proper stem cell differentiation. Regarding the hardware set up of the bioprinter, some future low-cost suggestions can include the integration of a UV crosslinking system (Galarraga et al., 2019), along with a temperature-controlled nozzle (Moncal et al., 2019). A more expensive integration, would be a pneumatic based extrusion system, which enables the simultaneous bioprinting of more than one bioinks due to less weight and more controllable extrusion pressure (Ning et al., 2020). Moreover, when bioprinting cells with low viscosity bioinks is required, new methods could be applied such as FRESH (Jiang et al., 2017) and CLASS (Mirdamadi et al., 2019). These setups will ultimately pave the way to more accurate bioprinting procedures with the aim to grow *in vitro* organ grafts of desired size and shape, derived from the patients’ own cells.

## Data Availability Statement

The datasets presented in this study can be found in online repositories. The names of the repository/repositories and accession number(s) can be found in the article/Supplementary Material.

## Ethics Statement

The animal study was reviewed and approved by the Achaia’s regional veterinary authority.

## Author Contributions

ST and KI conceived the idea and supervised the project. KI generated the CAD files and converted the 3D bioprinter. KI, SC, and RD conducted experiments and analyzed data. KK and GL conducted the isolation of pRGCs. KI and AK conducted experiments regarding the osteogenic differentiation of MSCs. GB and KI conducted the rheological analysis of the bioinks. ST, KI, SC, ZL, and DP provided materials and helped write the manuscript. All authors contributed to the article and approved the submitted version.

## Conflict of Interest

The authors declare that the research was conducted in the absence of any commercial or financial relationships that could be construed as a potential conflict of interest.

## Footnotes

1 https://www.tinkercad.com/

2 https://www.pronterface.com/

## Supplementary Material

Supplementary Video 1 Footage of 3D bioprinting process.

Supplementary Video 2 **(A)** Video illustration produced by showing all stacks from brightfield and fluorescence channels obtained from confocal microscopy on HeLa cells at day 3. Cells were immunostained using antibodies against Ki67 (red), α-tubulin (green) and Hoechst (blue). **(B)** 3D rotating projection of HeLa cells from day 3 **(A)** without brightfield.

Supplementary Video 3 **(A)** Video illustration produced by showing all stacks from brightfield and fluorescence channels obtained from confocal microscopy on HeLa cells at day 10. Cells were immunostained using antibodies against Ki67 (red), α-tubulin (green) and Hoechst (blue). **(B)** 3D rotating projection of HeLa cells from day 10 (Video S3a) without brightfield.

Supplementary Video 4 pRGCs neurosphere from day 15 (Supplementary Figure S2) shown using rotating 3D projection.
