# Supplementary material for: A Custom Ultra-Low-Cost 3D Bioprinter Supports Cell Growth and Differentiation
Source: Front Bioeng Biotechnol. 2020 Nov 4;8:580889. doi: 10.3389/fbioe.2020.580889 (PMC7676439; doi:10.3389/fbioe.2020.580889)
Supplement: Supplementary Video 1 — Footage of 3D bioprinting process. [file Presentation_1.zip › Supplementary Figures and Table.pdf]

Supplementary Material

1 Supplementary Figures and Tables

1.1 Supplementary Table

Table 1. Primer sequences used for RT-qPCR analysis in MSCs 3D bioprinted cultures.

Runx2F	CCGCACGACAACCGCACCAT
Runx2R	CGCTCCGGCCCACAAATCTC
OsteopontinF	TCACCATTCGGATGAGTCTG
OsteopontinR	ACTTGTGGCTCTGATGTTCC
Alkaline PhosphataseF	ATCGGAACAACCTGACTGACCCTT
Alkaline PhosphataseR	ACCCTCATGATGTCCGTGGTCAAT
Collagen Type 1F	TTCTCCTGGCAAAGACGGACTCAA
Collagen Type 1R	AGGAAGCTGAAGTCATAACCGCCA

1.2 Supplementary Figures

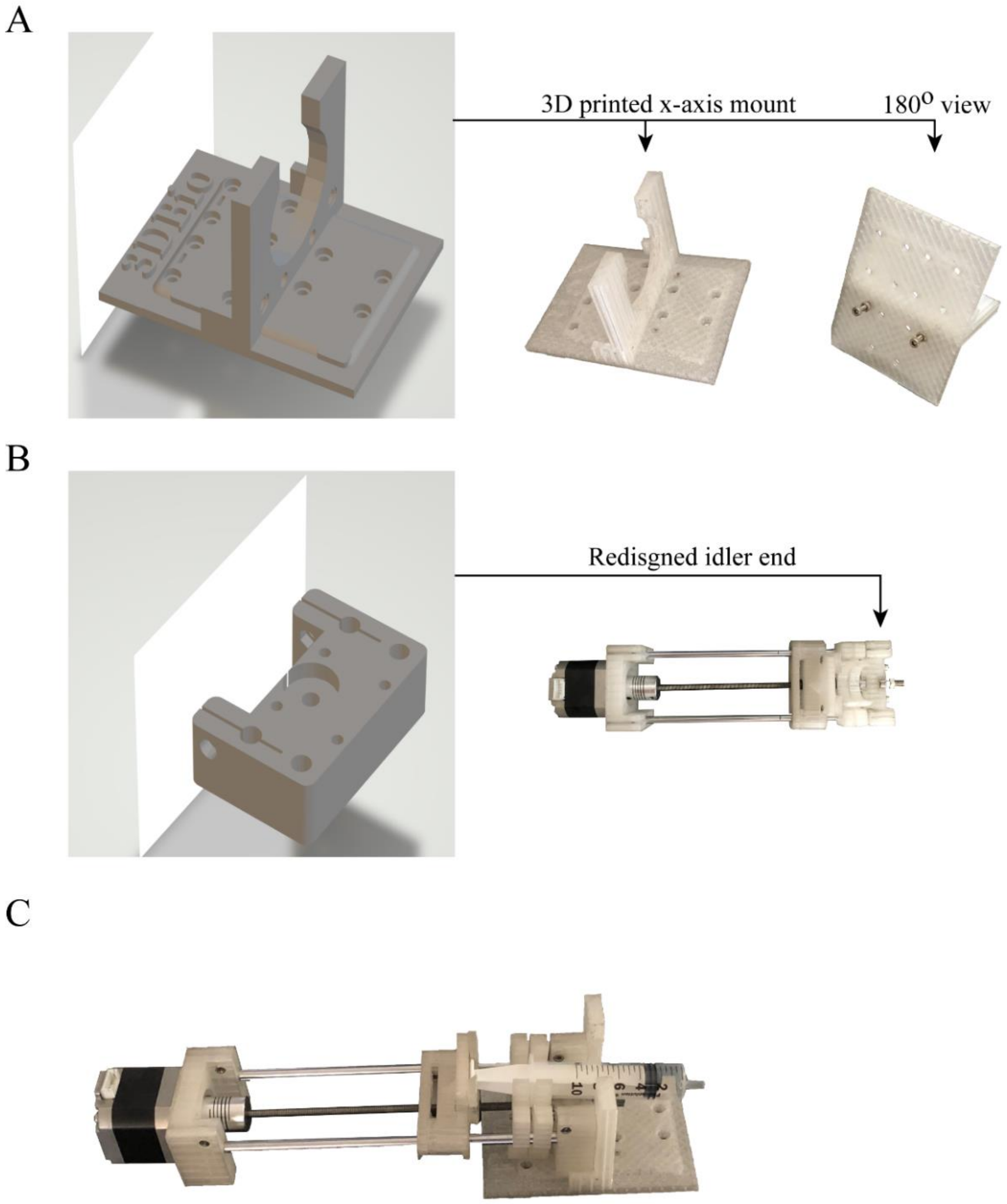

Supplementary Figure S1. Schematic of the bioprinter's conversion: A) 3D representation of our originally designed CAD file of the x-axis mount, along with photographs of the actual 3D printed part in two side views. B) 3D representation of the redesigned idler end and a photograph showing the assembled syringe pump unit. C) The syringe pump unit integrated with the x-axis mount.

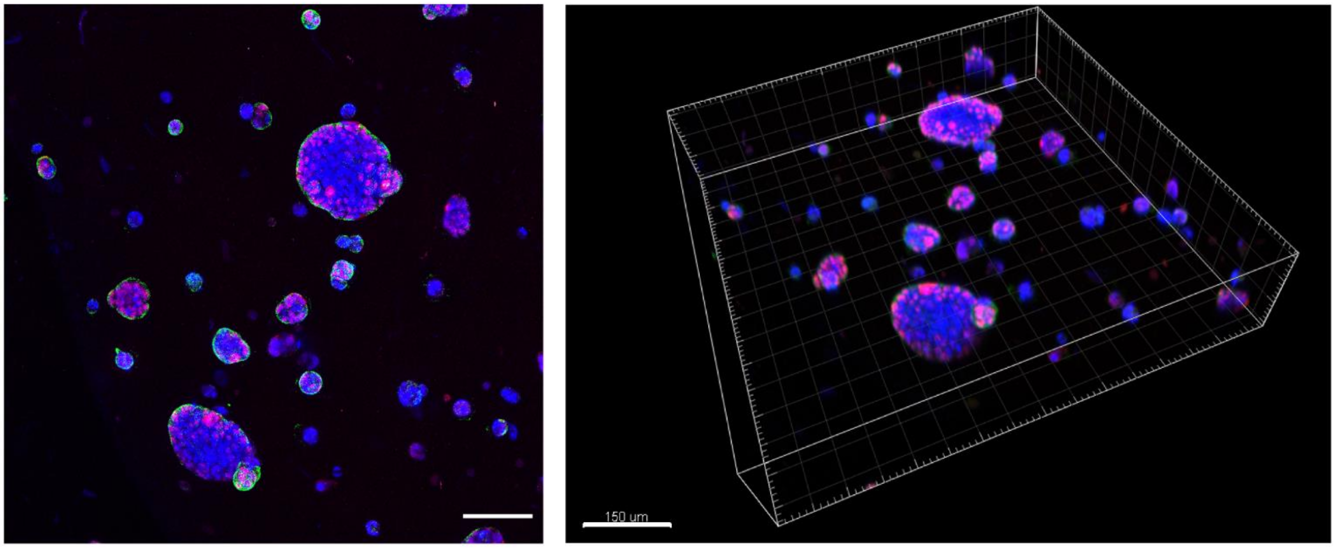

Supplementary Figure S2. Confocal maximal projection image and its 3D reconstruction, showing HeLa cells at day 15 in 3D bioprinted cultures. Cells were immunostained against Ki67 (red), α-tubulin (green) and Hoechst (blue). Scalebar represents 100 µm.

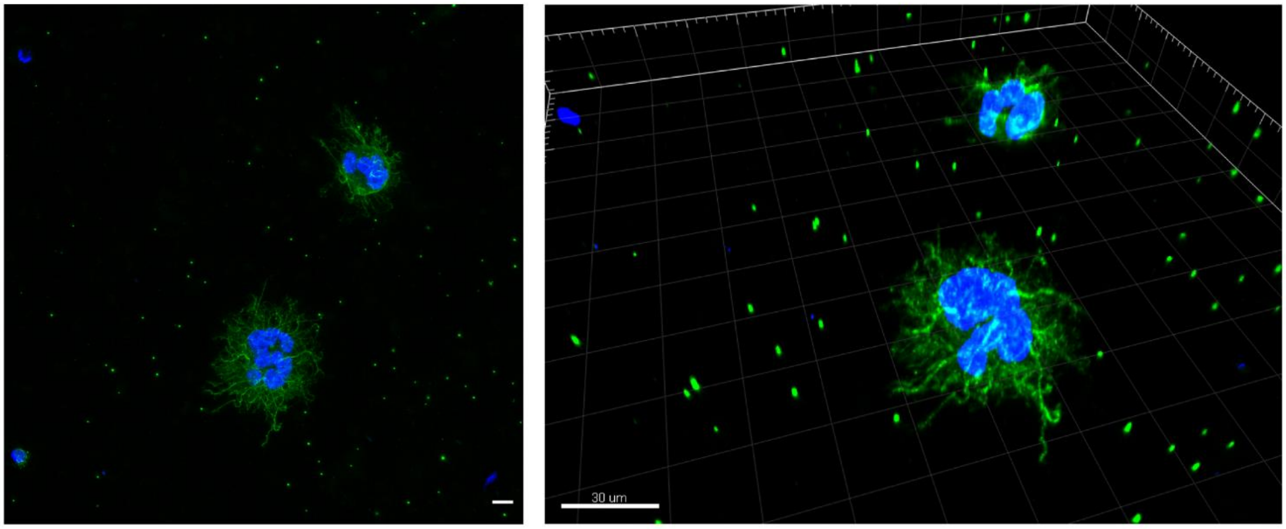

Supplementary Figure S3. Confocal maximal projection image and its 3D reconstruction, showing postnatal radial glial cells (pRGCs) at day 15 in 3D bioprinted cultures. Cells were immunostained using anti-α-tubulin (green) and Hoechst (blue). Scalebar represents 10 µm.

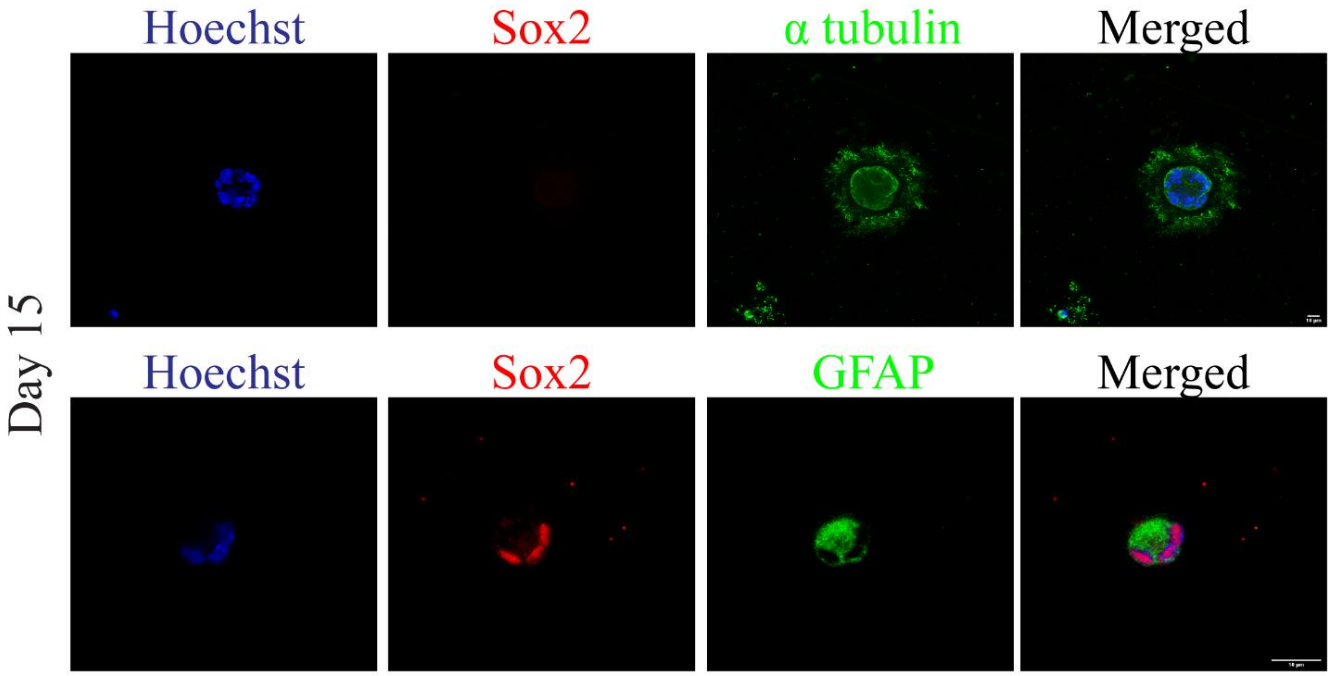

Supplementary Figure S4. Heterogeneity in cellular composition of 3D pRGCs cultures for 15 days. Neurospheres were subjected to immunofluorescent using specific antibodies that recognize (A): Sox2 (red), α tubulin (green) and (B): Sox2 (red), GFAP (green) and observed by confocal fluorescence microscopy. Nuclei were stained with Hoechst (blue). Scalebar represents 10μm.

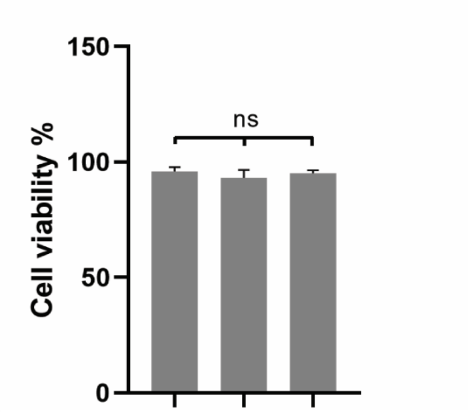

Supplementary Figure S5. Cell viability assessment prior and without been mixed within bioink [C (control)] and following incubation right after HeLa cells were mixed within the bioink consisting of alginate 1.8% and gelatin 3% for 30 min at 4°C and 37°C. No significant cell death was observed. All measurements were conducted in triplicate.

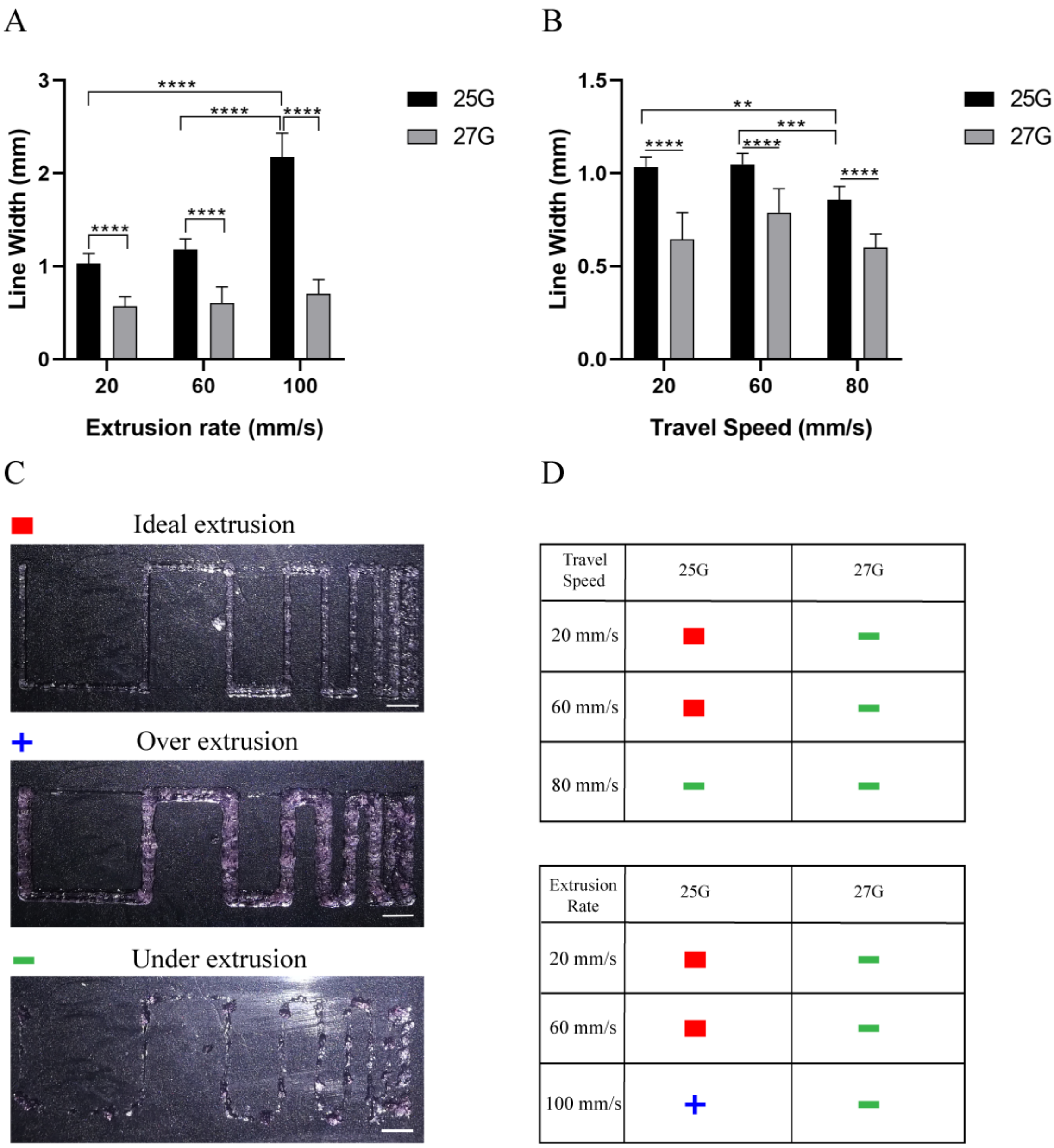

Supplementary Figure S6. Influence of printing parameters on bioprinting outcome. (A) Range of extrusion rate values and its influence in the printing outcome using 25G and 27G nozzles with a constant travel speed of 60mm/s. (B) Range of travel speed values and its influence in the printing outcome using 25G and 27G nozzles with a constant extrusion rate of 60 mm/s. (C) Representative images of printed structures showing an ideally extruded, an over extruded and an under extruded construct. (D) Classification of printing outcome at different extrusion rates, travel speed and nozzle diameters. The bioink used in this study was A2 G3. Scalebars represent 5 mm. All experiments were conducted in triplicate (n=3). Values are mean ± SD. *p<0.05, **p<0.01, ***p<0.001 and ****p<0.0001.
